# Urban–Rural Variations in Quality-of-Life in Breast Cancer Survivors Prescribed Endocrine Therapy

**DOI:** 10.3390/ijerph14040394

**Published:** 2017-04-07

**Authors:** Caitriona Cahir, Audrey Alforque Thomas, Stephan U. Dombrowski, Kathleen Bennett, Linda Sharp

**Affiliations:** 1 Division of Population Health Sciences, Royal College of Surgeons in Ireland, Dublin 2, Ireland; kathleenebennett@rcsi.ie; 2 Office of Planning & Analysis, University of California, Berkeley, CA 94720, USA; audrey@berkeley.edu; 3 Division of Psychology, University of Stirling, Stirling FK9 4LA, UK; s.u.dombrowski@stir.ac.uk; 4 Institute of Health and Society, Newcastle University, Newcastle NE1 7RU, UK; Linda.Sharp@newcastle.ac.uk

**Keywords:** urban, rural, quality of life, breast cancer, survivorship, endocrine therapy

## Abstract

The number of breast cancer survivors has increased as a result of rising incidence and increased survival. Research has revealed significant urban–rural variation in clinical aspects of breast cancer but evidence in the area of survivorship is limited. We aimed to investigate whether quality of life (QoL) and treatment-related symptoms vary between urban and rural breast cancer survivors prescribed endocrine therapy. Women with a diagnosis of stages I–III breast cancer prescribed endocrine therapy were identified from the National Cancer Registry Ireland and invited to complete a postal survey (*N* = 1606; response rate = 66%). A composite measure of urban–rural classification was created using settlement size, population density and proximity to treatment hospital. QoL was measured using the Functional Assessment of Cancer Therapy (FACT-G) and an endocrine subscale. The association between urban–rural residence/status and QoL and endocrine symptoms was assessed using linear regression with adjustment for socio-demographic and clinical covariates. In multivariable analysis, rural survivors had a statistically significant higher overall QoL (β = 3.81, standard error (SE) 1.30, *p* < 0.01), emotional QoL (β = 0.70, SE 0.21, *p* < 0.01) and experienced a lower symptom burden (β = 1.76, SE 0.65, *p* < 0.01) than urban survivors. QoL in breast cancer survivors is not simply about proximity and access to healthcare services but may include individual and community level psychosocial factors.

## 1. Introduction

More than 5 million women worldwide are living with breast cancer, almost half of whom reside in developed countries [1]. Each year in Ireland, approximately 2600 women are diagnosed with breast cancer and 660 women die from the disease. One in 10 women in Ireland will get breast cancer at some stage in their lives [2]. In recent years, with earlier diagnosis and better treatment options, breast cancer survival has increased steadily in most developed countries; it is now estimated to be 82% at 5 years [3,4]. After completing surgery and any chemotherapy or radiotherapy, the overwhelming majority of breast cancer patients do not have progressive or terminal disease [5]. They thus transition from a being a breast cancer patient to becoming a breast cancer survivor [6].

A critical part of the transition to survivorship care, for women with estrogen receptor positive (ER+) breast cancer, is the use of endocrine therapy. Five to ten years of adjuvant endocrine therapy is recommended to reduce the risks of recurrence and mortality in women with ER+ early breast cancer [7]. Previous research indicates that almost 75% of breast cancers are hormone receptor-positive [8]. Endocrine therapies are known for their adverse effects, which include reduced cognitive function, hot flashes, painful joints and vaginal dryness; these can have a negative impact on quality of life (QoL), adherence to endocrine therapy and, consequently survival [9,10,11]. Hence, despite significant therapeutic advances and improvements in survival at the population-level, some breast cancer survivors experience considerable physical and psychosocial dysfunction and reduced QoL [12].

The past decade has seen major growth in research investigating the diverse experiences, complex needs and QoL of breast cancer survivors [13]. However, there are a number of gaps in the knowledge base. For example, in relation to those on endocrine therapy, it is unclear why some survivors experience few adverse effects from their therapy, while others with similar disease and treatment may have many. More generally, it is likely that the characteristics of the communities in which breast cancer survivors live can have an effect on their survivorship outcomes; women who live in rural and urban areas may have different supportive care needs (because of differences in availability of services for example) and this may translate into differences in QoL. Research has revealed evidence of significant disparities in clinical aspects of breast cancer by area of residence with more advanced stage at diagnosis, lower treatment utilisation, poorer survival and higher mortality in women resident in rural areas [14,15,16]. However, evidence in the area of breast cancer survivorship is limited and findings are inconsistent. 

A small US study (*N* = 46) found that, one month post-chemotherapy, breast cancer survivors resident in more rural areas reported lower overall QoL, lower functional well-being and more breast cancer specific symptoms, than urban dwellers [17]. Similarly, in Europe, a large study in Germany (*N* = 1927) found that survivors in rural areas had worse QoL [15]. In contrast, in Poland, those who were resident in rural areas rated their social QoL higher than those in urban areas [18,19]. Meanwhile, in Australia a study of 600 survivors one year after breast cancer diagnosis, found that age-adjusted QoL among urban and rural survivors was similar [12]. None of these studies focused on survivors on endocrine therapy.

There is a need to further investigate associations between urban–rural residence and aspects of QoL in different settings where concepts of urbanisation and rurality may have different meanings and implications. Such research would help to establish whether there are disparities in survivors’ supportive care needs and, if so, enable the appropriate and effective development and delivery of health and supportive care services to all sectors of the breast cancer survivor population [20]. The aim of this study, therefore, was to investigate whether QoL and treatment-related symptoms vary between urban and rural survivors of breast cancer prescribed adjuvant endocrine therapy. 

## 2. Methods

### 2.1. Study Population

Women with breast cancer were identified in August 2015 from the National Cancer Registry Ireland (NCRI) database [21]. The NCRI records detailed demographic and clinical information for all incident cancers diagnosed in the population usually resident in Ireland. Completeness of registration is high, especially for breast cancer [22]. Eligibility criteria were (i) aged ≥18 years; (ii) had a diagnosis of stages I–III, estrogen (ER) or progesterone (PR) receptor positive breast cancer between 1 July 2009 and 30 June 2014; (iii) received tumour directed surgery; (iv) were prescribed adjuvant endocrine therapy (selective estrogen receptor modulator, SERM; aromatase inhibitor, AI) within one year of their breast cancer diagnosis and for no more than 5 years before the study start date; and (v) were alive. Women were excluded if they had previously been diagnosed with another invasive cancer other than non-melanoma skin cancer. 

Each potentially eligible woman’s details were screened by their General Practitioner (GP) to confirm that there was no medical or other reason that would make it inappropriate to contact them about the study. The remaining eligible women were invited, by post, to take part in the study and self-complete a questionnaire [21]. Ethical approval was granted by the Irish College of General Practitioners. All participants provided informed consent to participate in the study.

### 2.2. Outcome Measures QoL

QoL was measured using the Functional Assessment of Cancer Therapy (FACT-G) [23]. FACT-G is a well validated multi-dimensional self-report questionnaire that assesses four primary domains of QoL; physical (PWB: seven items), social and family (SFWB: seven items), emotional (EWB: six items) and functional well-being (FWB: seven items). It asks about the past week and uses 5-point Likert-type response categories ranging from 0 = ‘not at all’ to 5 = ‘very much’ [23]. It has good psychometric properties, discriminates well between clinically distinct groups, and is responsive to change [24,25]. It is also validated for use in different countries and with rural populations [26,27]. The individual domain scores were calculated using the pre-defined scoring programme; as recommended, where participants had to have answered at least half of the questions in a domain to be included in the subscale score for that domain [28]. A 19-item endocrine subscale (ES) was also included which measures endocrine symptoms and adverse effects of endocrine therapy [29,30]. The endocrine subscale uses the same 5-point Likert-type response categories and scoring method as the FACT-G. The endocrine subscale score is added to the FACT-G to give an overall QoL score (FACT-ES) for women with breast cancer prescribed endocrine therapy [29,30]. A higher overall QoL score (FACT-ES) and higher individual domain scores and endocrine subscale scores indicate higher/better QoL [28].

### 2.3. Urban–Rural Measure

A number of health-related studies have indicated that the use of a single indicator to identify urban–rural areas does not adequately capture the range of urban and rural area types and suggest the use of multiple measures to reduce the probability of misclassification [31]. A composite measure of urban–rural classification was created using three indicators; settlement size, population density and proximity to treatment hospital. These indicators have all been used previously as indicators in urban–rural classifications and take into account the range of settlement and area types in Ireland [31,32]. The use of a composite measure gives a more complete measure of urbanicity and rurality, rather than privileging one type of measurement over another [32]. Participants who were classified as rural for at least two of the three component indicators were classified as rural for the composite measure [32].

Settlement size was measured as the self-reported area size where the participant was living at the time of the questionnaire completion. In line with the Irish Census Office definition of rural, participants who responded “Village” and “Open country” were classified as rural and those who responded “Dublin city/county”, “City other than Dublin” and “Town (1500+)” were classified as urban [33]. Population density of the area the participant lived at the time of questionnaire completion was classified as rural if “<1 person per hectare” and urban if “≥1 person per hectare” based on 2006 census small-area populations. This urban–rural classification has been used in Ireland in previous studies [32,34]. Proximity to treatment hospital was measured as the distance from the participant’s residence at diagnosis to the main treatment hospital. The main treatment hospital was defined as where the participant had their breast cancer surgery or other treatments/procedures (e.g., chemotherapy, radiotherapy), or both and was obtained from the NCRI database. Participants were grouped into three approximately equal-sized tertiles based on this distance. The last tertile of distance from the treatment hospital (≥60 km; approximately 75 min travel time) was classified as rural, while the two closest tertiles (<60 km) were classified as urban [31,32].

### 2.4. Covariates

Socio-demographic and clinical variables identified as predictors of QoL in the literature were included as covariates [35]. Socio-demographic covariates included women’s self-reported age, relationship status, education level, occupational status, socioeconomic status and smoking status. Women who reported being married, cohabiting or in a long-term relationship with a partner were classified as being in a relationship. Socioeconomic status was measured using a census-based deprivation score (five levels ranging from least to most deprived) [36]. Women were also asked whether they had a medical card at the time of their breast cancer diagnosis. A medical card provides individuals with free or substantially-subsidised health care and prescription medications and eligibility is means-tested; medical card status is therefore considered a proxy for socioeconomic status [37]. Smoking status at diagnosis was obtained from the NCRI database. Clinical information available from the NCRI database included receipt of chemotherapy and radiotherapy. Women were classified into four treatment groups: (i) chemotherapy only; (ii) radiotherapy only; (iii) chemotherapy and radiotherapy and; (iv) no chemotherapy or radiotherapy.

### 2.5. Statistical Analysis

Descriptive statistics including means (standard deviation, SD), medians (inter-quartile range, IQR) and proportions, were calculated for all socio-demographic, clinical, QoL and endocrine symptoms (FACT-ES) variables. Urban–rural differences between these variables were examined using chi-square tests for categorical variables and non-parametric Wilcoxon–Mann–Whitney tests for continuous variables (participants’ age, QoL and endocrine symptoms).

The association between the composite urban–rural variable and overall QoL (FACT-ES) and for each individual domain and endocrine symptoms were assessed using linear regression analysis with adjustment for the socio-demographic and clinical covariates described above and using robust standard errors. The use of robust standard errors (*sandwich estimator*) controls for mild violation of the homoskedasticity and normality distribution assumption in linear regression [38]. The data was analysed using Stata Version 14.0 (StataCorp, College Station, TX, USA).

## 3. Results

### 3.1. Study Population

Of the 1606 (response rate = 66%) breast cancer survivors who completed the questionnaire, 1568 (98%) had complete urban–rural data and were included in the analysis. The mean age of these women was 60 years (SD = 10.2, range = 34–86 years); the majority were married or in a long-term relationship (*N* = 1175, 73.4%) and almost half (720, 45.3%) had completed third level (degree) or post-graduate education. There were no significant differences in age between responders and non-responders but the proportion of women married was significantly higher in the respondent group (73%) than in the non-respondent group (61%, *p* value < 0.01).

In total, 870 (55.5%) participants were classified as urban and 698 (44.5%) were classified as rural. Table 1 presents the socio-demographic and clinical characteristics of the survivors by urban and rural residence. A statistically significant higher proportion of rural participants were married or in a long-term relationship compared with urban participants (*p* < 0.01). A higher proportion of urban participants were resident in more affluent areas (*p* < 0.01) and were former smokers compared to rural participants (*p* < 0.05). There were no statistically significant clinical differences between rural and urban participants (Table 1). 

### 3.2. QoL and Endocrine Symptoms

Table 2 presents summary statistics for overall QoL (FACT-ES), the individual QoL domains and the endocrine subscale by urban–rural residence. In these unadjusted analyses, breast cancer survivors living in rural areas reported better overall (Wilcoxon–Mann–Whitney *z-*score = −2.69, *p* < 0.01), physical (*z-*score = −2.10, *p* < 0.05), emotional (*z-*score = −3.57, *p* < 0.01) and functional (*z-*score = −3.17, *p* < 0.01) QoL than women living in urban areas. Social domain scores and endocrine symptoms did not significantly differ by urban–rural residence. 

### 3.3. Multivariable Analysis: Associations between Urban–Rural Variation and QoL and Endocrine Symptoms

Table 3 presents the association between the FACT-ES, the individual QoL domains and the endocrine subscale and urban–rural residence, following adjustment for socio-demographic and clinical covariates. In these multivariable analyses, breast cancer survivors living in rural areas had a statistically significant higher overall QoL (β = 3.81, standard error (SE) 1.30, *p* < 0.01) and emotional QoL (β = 0.70, SE 0.21, *p* < 0.01) and a lower endocrine symptom burden (β = 1.76, SE 0.65, *p* < 0.01) than urban survivors. Physical and functional QoL were no longer significantly associated with living in rural areas.

In terms of socio-demographic and clinical covariates, increasing age (β = 0.59, SE 0.09, *p* < 0.01), having never smoked (β = 4.82, SE 2.00, *p* < 0.05) and receipt of radiotherapy only (without chemotherapy) (β = 4.96, SE 2.53, *p* < 0.05) were associated with a significantly higher overall QoL. Inability to work due to sickness or disability (β = −22.44, SE 2.66, *p* < 0.01) was significantly associated with a lower overall QoL (Table 3). Women who were married or in a long-term relationship had a significantly higher social (β = 1.59, SE 0.42, *p* < 0.01) and functional QoL (β = 1.06, SE 0.39, *p* < 0.01) but experienced a greater endocrine symptom burden (β = −2.73, SE 0.76 *p* < 0.01) (Table 3).

## 4. Discussion

In this, the first study of urban–rural variations in QoL in survivors of stages I–III breast cancer prescribed endocrine therapy, those living in rural areas had significantly higher emotional and overall QoL and experienced a lower endocrine symptom burden than those living in urban areas, after controlling for socio-demographic and clinical variables. The difference in QoL between rural and urban breast cancer survivors was approximately four points and, therefore, would be considered clinically significant. The minimal important difference (MID) for interpreting group differences or changes in QoL over time for FACT breast cancer scales is estimated to be in the range of 3–8 points [39,40].

These intriguing findings are inconsistent with previous research [41]. The majority of studies have reported lower QoL in cancer survivors in rural settings and have found that rural patients have higher needs in the domains of physical/daily living and psychological morbidity and are more likely to experience distress, high levels of depression and hopelessness/helplessness, as well as stigmatisation [17,41]. However, the findings are consistent with a study of QoL in urban and rural settings in head and neck cancer survivors in Ireland. In that study, rural survivors reported higher physical and emotional QoL than urban survivors [32]. The findings are also consistent with a study of the general population in Ireland which found better psychosocial well-being in rural populations [42].

Rural and urban life is not homogenous across countries and this may help explain differences between countries. Traditionally, rural living is often perceived as advantageous in terms of offering greater space and a slower pace of life than urban living but disadvantageous in terms of poorer access to health services and healthcare professionals [31,43]. Our findings suggest that access to services and professionals may not be a major driver of survivorship outcomes, at least in Ireland. Clearly, many different regional, community and individual patient factors may influence QoL for breast cancer survivors [17]. Understanding the reasons for greater well-being in breast cancer survivors in rural areas in Ireland requires identifying the major influences on QoL in women resident in different areas in the early stages following breast cancer treatment completion. 

In terms of explaining our findings, it is possible that urban and rural woman differ in the ways in which they cope with endocrine side-effects or in the impact of their coping strategies on their QoL [44]. The finding of higher emotional well-being in rural women may be related to coping, specifically an increased ability to cope with the stress and severity of their breast cancer and its treatment. A study of psychological adjustment in rural and urban breast cancer survivors found that a belief that their decisions and life are controlled by environmental factors which they cannot influence (external locus of control) had a negative impact on the psychological well-being of urban breast cancer patients, but this was not true for rural patients [45]. Studies have indicated that rural women with breast cancer tend to adopt a “positive attitude” as a coping strategy in response to their cancer [46]. Coping strategies such as “active coping” and “positive reinterpretation” have been found to be negatively associated with depression in rural breast cancer patients undergoing radiotherapy but not in their urban counterparts [44]. There is also evidence that people living in rural areas tend to be more stoic and resilient about their health and differ in their expectations and demands of the health services than their urban counterparts [47,48].

Social support has been shown to be a strong positive predictor of QoL in breast cancer survivors [35]. Women in a relationship experienced a greater endocrine treatment burden but had significantly higher social and functional QoL domain scores than their single counterparts. Previous research has found that socially isolated women have a higher risk of mortality after a breast cancer diagnosis and this is likely due to a lack of beneficial caregiving from friends, relatives and adult children [49]. Previous research has also indicated that among breast cancer survivors, the most important source of support is often not the women’s spouses but other members of their social network [49]. There is evidence, in Ireland, that urban dwellers are more socially isolated compared to rural dwellers [50]. In a national Irish lifestyle survey, those living in rural areas reported a greater ability to get both practical help and personal support from neighbours than those living in urban areas [51]. A study of head and neck cancer survivors in Ireland also found that having problems getting support from neighbours was associated with having unmet needs [52].

Geographic distance to professional clinical and psychological support has been cited as a reason for poorer QoL in rural cancer populations. In Ireland, 7% of rural patients live within walking distance of their General Practitioner (GP) compared to 89% of urban dwellers and the longest average travel times occur in the most deprived rural areas [53]. However other characteristics of rural communities may actually be of benefit to women coping with cancer including their local community services and their social networks [17]. It is feasible that as breast cancer survivorship care has become predominantly outpatient based care, breast cancer survivors in the rural setting may have developed local social networks, with network members providing informal emotional support previously provided by healthcare professionals [49]. Informal supports in rural communities have been reported to be highly effective in assisting people through a health-related crisis [54].

In terms of socio-demographic and clinical covariates, increasing age, having never smoked and receipt of radiotherapy only were associated with greater well-being, while inability to work (due to sickness/ill-health/unemployment) had a negative influence on QoL. These factors have all been identified previously as predictors of QoL in breast cancer survivors [35]. Receipt of chemotherapy has been shown to be a statistically significant predictor of a poor current quality of life [55]. Increasing age was also associated with experiencing less endocrine symptoms. Adverse effects of endocrine therapy are more common in older woman but studies have reported no impact on their QoL scores, possibly due to older women being less likely to report complaints than younger women [30,56].

This is the first study of urban–rural variations in QoL among breast cancer survivors prescribed endocrine therapy. The sampling frame was a high-quality population-based cancer registry. Although the response rate was 66%, we cannot exclude the possibility that respondents had better (or worse) QoL than non-respondents but, unless response was differential by area of residence, this should not affect the urban–rural comparisons. The cross-sectional design means we not know whether temporal trajectories of QoL vary between urban and rural survivors. While we adjusted for a range of socio-demographic and clinical covariates, we lacked information on other potentially important influences on QoL (such as coping styles and social support). We also did not consider the type of endocrine therapy women were prescribed and whether or not they were adherent to their therapy. Previous research has found that many women do not take their endocrine therapy as recommended; rates of non-adherence range between 38% to 60% in routine clinical settings at 5 years [57,58].

Given our findings, a more thorough investigation of the psychosocial influences on QoL is needed in order to understand the differences in QoL in breast cancer survivors in urban and rural settings. This includes an exploration of individual coping skills, self-efficacy and resilience in urban and rural breast cancer survivors who are prescribed endocrine therapy. Future research should also document and evaluate both the formal and informal support services available and accessed by urban and rural breast cancer survivors, as well as the quality and type of support (e.g., instrumental, emotional).

## 5. Conclusions

In conclusion, this large study of breast cancer survivors prescribed endocrine therapy found that those living in rural areas had significantly higher emotional and overall QoL and experienced a lower endocrine symptom burden than those living in urban areas. These findings suggest that QoL in breast cancer survivors is not simply about access and proximity to health care services and professionals. In order to develop interventions and services tailored to the specific needs of these two populations, future research needs to identify both the individual and community level psychosocial factors influencing QoL in breast cancer survivors in rural settings and whether these factors can be transferred to an urban setting.

## Figures and Tables

**Table 1:** Socio-demographic and clinical characteristics of urban and rural survivors of stages I–III breast cancer prescribed endocrine therapy.

	Urban (*N* = 870)	Rural (*N* = 698)	*p*-Value ^a^
	*N* (%)	*N* (%)	
***Socio-demographics***			
Age—mean (SD)	60.4 (10.3)	59.7 (10.1)	*p* = 0.17
*Marital status*			
Not in a relationship	266 (30.7)	152 (21.8)	*p* < 0.01 *
Married/long-term relationship ^b^	599 (69.3)	545 (78.2)	
*Education*			
Primary	110 (12.8)	95 (13.7)	*p* = 0.74
Secondary	362 (42.1)	279 (40.3)	
Third level or post-graduate	388 (45.1)	318 (46.0)	
*Occupational status*			
Employed (employee/self-employed)	313 (36.4)	271 (39.0)	*p* = 0.08
Looking after family/home	168 (19.6)	165 (23.7)	
Retired from employment	248 (28.9)	169 (24.3)	
Unable to work (sickness/disability)	86 (10.0)	63 (9.1)	
Other ^c^	44 (5.1)	27 (3.9)	
*Deprivation*			
1 (least deprived)	205 (23.6)	62 (8.9)	*p* < 0.01 *
2	196 (22.5)	152 (21.8)	
3	143 (16.4)	197 (28.2)	
4	157 (18.1)	180 (25.8)	
5 (most deprived)	169 (19.4)	107 (15.3)	
*Smoking status at diagnosis*			
Current	124 (14.5)	100 (14.6)	*p* < 0.05*
Former	145 (16.9)	78 (11.3)	
Never	460 (53.7)	400 (58.2)	
Unknown	128 (14.9)	109 (15.9)	
*Medical card* ^d^			
Yes	276 (32.0)	288 (32.7)	*p* = 0.09
No	587 (68.0)	469 (67.3)	
***Clinical***			
*Chemotherapy and/or Radiotherapy*			
Chemotherapy only	82 (9.4)	66 (9.5)	*p* = 0.25
Radiotherapy only	330 (37.9)	252 (36.1)	
Chemotherapy and radiotherapy	372 (42.8)	327 (46.9)	
No chemotherapy or radiotherapy	86 (9.9)	53 (7.6)	

* *p* < 0.05; ^a^ Wilcoxon–Mann–Whitney test; ^b^ Long-term relationship—cohabiting or in a stable relationship; ^c^ Other—unemployed, student (undertaking training/education); ^d^ Medical card provides individuals with free or substantially-subsidised health care and prescription medications and eligibility is means-tested, medical card status is therefore considered a proxy for socioeconomic status; SD: Standard deviation.

**Table 2:** Median (with IQRs) for overall QoL domain scores and endocrine symptoms for urban and rural survivors of stages I–III breast cancer.

	All Participants		Urban (*N* = 870)			Rural (*N* = 698)			Urban–Rural Comparison	
QoL	Median	IQR	*N*	Median	IQR	*N*	Median	IQR	*z*-Score	*p*-Value
Overall QoL (FACT-ES)	124	106–140	804	123	104–138	646	126	107–143	−2.69	<0.01 *
Physical domain	24	21–27	845	24	20–27	681	25	21–27	−2.10	<0.05 *
Social domain	22	17–26	844	22	17–26	677	22	17–26	−0.56	0.58
Emotional domain	20	17–23	838	20	17–22	671	20	18–23	−3.57	<0.01 *
Functional domain	21	17–26	843	21	17–25	672	22	18–26	−3.17	<0.01 *
Endocrine symptoms	40	29–48	845	39	29–47	681	41	30–49	−1.90	0.06

IQR: inter-quartile range; QoL: quality of life; FACT-ES: Functional Assessment of Cancer Therapy, endocrine subscale; * Wilcoxon–Mann–Whitney test: A higher overall QoL (FACT-ES), individual domain score or endocrine symptom score indicates a higher/better QoL (or lower symptom burden).

**Table 3:** Unadjusted and adjusted coefficients (SE) for FACT-ES, individual domains and endocrine symptoms by urban–rural, socio-demographic and clinical factors ^a^.

	Overall QoL (FACT-ES)		Physical		Social		Emotional		Functional		Endocrine Symptoms	
	Unadjusted β (SE)	Adjusted β (SE)	Unadjusted β (SE)	Adjusted β (SE)	Unadjusted β (SE)	Adjusted β (SE)	Unadjusted β (SE)	Adjusted β (SE)	Unadjusted β (SE)	Adjusted β (SE)	Unadjusted β (SE)	Adjusted β (SE)
***Urban*–*rural*** Rural (vs. urban)	3.85 (1.36) *	3.81 (1.30) *	0.48 (0.25) *	0.36 (0.24)	0.25 (0.33)	0.11 (0.35)	0.76 (0.20) *	0.70 (0.21) *	0.81 (0.32) *	0.53 (0.32)	1.37 (0.70) *	1.76 (0.65) *
***Socio-demographic*** Age (at survey completion- continuous)	0.70 (0.06)	0.59 (0.09) *	0.07 (0.01) *	0.06 (0.02) *	0.05 (0.02) *	0.06 (0.02) *	0.07 (0.01) *	0.05 (0.01) *	0.01 (0.02)	0.03 (0.02)	0.49 (0.03) *	0.40 (0.05) *
*Marital status (vs. no relationship) ^b^*												
Married/long-term relationship	−1.61 (1.54)	0.10 (1.53)	−0.17 (0.27)	−0.11 (0.28)	1.36 (0.39) *	1.59 (0.42) *	−0.22 (0.23)	−0.06 (0.24)	1.30 (0.37) *	1.06 (0.39) *	−4.73 (0.77) *	−2.73 (0.76) *
*Education (vs. primary)*												
Secondary	−3.79 (2.16)	−0.36 (2.23)	0.18 (0.37)	0.24 (0.41)	−0.80 (0.53)	−0.61 (0.57)	−0.26 (0.33)	0.05 (0.36)	0.56 (0.54)	0.04 (0.57)	−2.95 (1.09) *	−0.12 (1.11)
Third level or post-graduate	−6.07 (2.13) *	−1.55 (2.44)	−0.66 (0.37)	−0.60 (0.46)	−0.83 (0.51)	−0.47 (0.60)	−0.56 (0.32)	−0.11 (0.38)	1.16 (0.52) *	0.32 (0.60)	−4.89 (1.08) *	−1.00 (1.23)
*Occupational status (vs. employed)*												
Looking after family/home	5.84 (1.70) *	1.16 (1.88)	0.71 (0.29) *	0.09 (0.33)	0.57 (0.44)	0.02 (0.49)	0.46 (0.26)	0.02 (0.30)	−0.05 (0.39)	0.11 (0.41)	4.55 (0.88) *	1.09 (0.97)
Retired from employment	9.71 (1.60) *	0.99 (1.99)	0.64 (0.27) *	−0.23 (0.35)	0.72 (0.41)	0.02 (0.55)	1.09 (0.24) *	0.26 (0.32)	−0.46 (0.39)	−0.23 (0.49)	7.57 (0.83) *	0.52 (1.01)
Unable to work (sickness or disability)	−23.24 (2.24) *	−22.44 (2.66) *	−5.04 (0.54)	−4.97 (0.55) *	−1.09 (0.57)	−1.08 (0.61)	−2.32 (0.42)	−2.17 (0.43) *	−6.16 (0.52) *	−5.43 (0.57) *	−8.44 (1.17) *	−8.88 (1.31) *
Other ^c^	−6.48 (3.11) *	−6.34 (3.41)	−0.92 (0.61)	−0.94 (0.64)	0.96 (0.95)	−1.26 (0.96)	−1.18 (0.56) *	−1.20 (0.54) *	−2.34 (0.82) *	−1.71 (0.87) *	−1.24 (1.59)	−1.11 (1.74)
*Deprivation (vs. least deprived)*												
2	1.84 (2.16)	0.70 (2.04)	0.79 (0.41)	0.62 (0.38)	−0.52 (0.53)	−0.53 (0.53)	0.05 (0.34)	0.01 (0.33)	0.32 (0.51)	0.45 (0.49)	1.51 (1.08)	0.69 (1.02)
3	−0.17 (2.18)	−0.93 (2.16)	0.31 (0.42)	0.25 (0.40)	−0.39 (0.53)	−0.34 (0.55)	0.31 (0.35)	0.25 (0.34)	−0.01 (0.53)	0.24 (0.52)	−0.15 (1.13)	−0.82 (1.07)
4	0.60 (2.19)	−0.37 (2.12)	0.44 (0.41)	0.31 (0.40)	−0.26 (0.53)	−0.29 (0.56)	0.16 (0.34)	0.07 (0.34)	0.38 (0.51)	0.71 (0.51)	0.41 (1.12)	−0.52 (1.07)
5	−1.10 (2.29)	−0.34 (2.26)	−0.29 (0.45)	−0.21 (0.45)	0.12 (0.55)	0.38 (0.58)	−0.04 (0.37)	0.16 (0.37)	−0.33 (0.55)	0.50 (0.55)	−0.19 (1.20)	−0.90 (1.14)
*Smoking status (vs. current)*												
Former	4.72 (2.96)	1.26 (2.59)	1.02 (0.49) *	0.55 (0.48)	0.15 (0.63)	−0.21 (0.65)	0.40 (0.42)	0.02 (0.42)	0.77 (0.58)	0.20 (0.58)	5.67 (1.12) *	3.08 (1.00) *
Never	9.60 (1.96) *	4.82 (2.00) *	1.42 (0.40) *	0.71 (0.37)	0.77 (0.50)	0.48 (0.52)	1.09 (0.34) *	0.63 (0.34)	1.35 (0.47) *	0.38 (0.48)	2.99 (1.37) *	1.26 (1.29)
Unknown	7.50 (2.46) *	3.99 (2.38)	1.19 (0.48) *	0.74 (0.45)	0.19 (0.64)	−0.03 (0.66)	1.05 (0.41) *	0.71 (0.41)	0.90 (0.59)	0.34 (0.58)	4.80 (1.34) *	2.62 (1.18)
*Medical card (vs. Yes)*												
No ^d^	0.47 (1.45)	2.50 (1.58)	0.43 (0.27)	0.45 (0.30)	0.14 (0.36)	0.12 (0.42)	0.31 (0.32)	0.56 (0.26) *	1.92 (0.35) *	1.34 (0.39) *	−2.64 (0.74) *	−0.04 (0.79)
***Clinical*** *Chemotherapy and/or radiotherapy (vs. none)*												
Chemotherapy only	−5.10 (3.06)	0.33 (3.24)	−1.08 (0.55) *	−0.30 (0.58)	0.74 (0.75)	1.13 (0.83)	−0.84 (0.46)	−0.39 (0.52)	0.28 (0.71)	0.98 (0.78)	−3.06 (1.58) *	0.05 (1.57)
Radiotherapy only	5.20 (2.43) *	4.96 (2.53) *	0.48 (0.44)	0.48 (0.43)	1.58 (0.60) *	1.49 (0.69) *	0.78 (0.37) *	0.79 (0.41)	1.41 (0.57) *	1.53 (0.66) *	1.20 (1.26)	0.97 (1.22)
Chemotherapy and radiotherapy	−0.83 (2.38)	3.88 (2.57)	−0.32 (0.43)	0.37 (0.45)	1.51 (0.59) *	1.67 (0.68) *	−0.41 (0.36)	0.04 (0.42)	0.66 (0.56)	0.97 (0.64)	−2.26 (1.24)	1.15 (1.26)

β: coefficient, SE: standard error; * *p* < 0.05; ^a^ Multicollinearity was assessed by calculating the variance inflation factor (VIF) for each independent variable (VIF < 10; mean VIF = 1.72); ^b^ Long-term relationship—cohabiting or in a stable relationship; ^c^ Other—unemployed, student (undertaking training/education); ^d^ Medical card provides individuals with free or substantially-subsidised health care and prescription medications and eligibility is means-tested, medical card status is therefore considered a proxy for socioeconomic status.
